# EARLY-LIFE SOCIOECONOMIC POSITION AND LATER-LIFE COGNITIVE FUNCTIONING: A META-ANALYSIS

**DOI:** 10.1093/geroni/igad104.0872

**Published:** 2023-12-21

**Authors:** Hanamori Skoblow, Francisco Palermo, Christine Proulx

**Affiliations:** University of Missouri, Columbia, Missouri, United States; University of Missouri, Columbia, Missouri, United States; University of Vermont, Burlington, Vermont, United States

## Abstract

A life course perspective on cognitive aging suggests that experiences early in life may be linked to later performance on cognitive tests. A growing literature shows that childhood socioeconomic position (SEP) is positively associated with later-life cognitive functioning. To identify the strength of this association and potential moderating variables, we used meta-analytic techniques to identify the magnitude of the association between early-life (aged 0-17) SEP and later-life (aged 50+) cognitive functioning. After systematically searching PsycINFO, Scopus, and ScienceDirect, 30 studies representing 39 samples and 223,381 participants met the inclusion criteria. Extracted data included participant and study design characteristics, construct operationalization and measures, and effect sizes. The results of the meta-analysis revealed a significant correlation between childhood SEP and later-life cognitive functioning, whether measured as an overall effect size (r = .179, 95% CI = [.156, .201]), global performance (r = .206, 95% CI = [.157, .255]), verbal episodic memory (r = .160, 95% CI = [.132, .188]), verbal fluency (r = .203, 95% CI = [.168, .237]), or processing speed (r = .146 (95% CI = .108, .184]), but not inhibition (r = .146, 95% CI = [-.150, .418]). Subgroup analyses revealed that, in some domains, the correlation was weaker with an older mean sample age. Correlations did not vary by study design (i.e., prospective or retrospective) or journal impact factor. These results provide important evidence for the promotion of policies that buffer children from economic hardship, as one’s cognitive performance in later life begins far earlier than older adulthood.

